# Development of a prognostic nomogram for metastatic pancreatic ductal adenocarcinoma integrating marital status

**DOI:** 10.1038/s41598-022-11318-1

**Published:** 2022-05-03

**Authors:** Xiang Ma, Junlong Guo, Cuiting Zhang, Jinfeng Bai

**Affiliations:** 1 Yunnan Caner Hospital, The Third Affiliated Hospital of Kunming Medical University, Kunming, 650118 China; 2 Kunming Medical University, Kunming, China

**Keywords:** Cancer, Oncology

## Abstract

Previous studies have shown that marital status can affect the overall survival (OS) of cancer patients yet its role in metastatic pancreatic ductal adenocarcinoma (mPDAC) remains unclear. This study aimed to explore the impact of marital status on the OS of mPDAC patients and to construct a prognostic nomogram to predict OS outcomes. Data from patients diagnosed with mPDAC were obtained from the Surveillance, Epidemiology, and End Results database between 1973 and 2015. The patients were randomized into primary and validation cohorts. Kaplan–Meier survival analysis was performed to compare differences in survival depending on marital status. Univariate and multivariate analyses were conducted to identify independent prognostic factors and a nomogram was established based using Cox regression analyses. Validation of the prognostic nomogram was evaluated with a calibration curve and concordance index (C-index). Our data showed significant differences in the OS of mPDAC patients with different marital status by Kaplan–Meier analysis (*P* < 0.05). Univariate and multivariate analyses confirmed that marital status was an independent OS-related factor in mPDAC patients. Based on the multivariate models of the primary cohort, a nomogram was developed that combined marital status, age, grade, tumor size, surgery of primary site, surgery of lymph node and metastatic. The nomogram showed that marital status had a moderate influence on predicting the OS of mPDAC patients. Moreover, the internally and externally validated C-indexes were 0.633 and 0.619, respectively. A calibration curve confirmed favorable consistency between the observed and predicted outcomes. Marital status was identified as an independent prognostic factor for OS of mPDAC patients and is a reliable and valid parameter to predict the survival of patients with mPDAC. This prognostic model has value and may be integrated as a tool to inform decision-making in the clinic.

## Introduction

Pancreatic cancer (PC) is the fourth most common cause of cancer-related deaths in the world^1,2^. Whilst improvements have been made in the treatment of PC, it continues to have poor outcomes with an extremely low 5 years overall survival of < 5%^3^. Most PC patients present with advanced or unresectable disease due to difficulties associated with early detection^4,5^. Pancreatic ductal adenocarcinoma (PDAC) is the most common type of PC in which most patients present with symptomatic and surgically unresectable disease. There is an urgent need for the development of improved treatments and predictive tools to inform clinical decision making for patients with metastatic pancreatic ductal adenocarcinoma (mPDAC).

In the new global economy, socio-economic and demographic variables have a critical role in the outcomes for patients with mPDAC. Marital status is a crucial component in determining disease stage and grade, as well as recurrence after treatment. Studies have shown that married couples receive better social support and have lower levels of chronic stress that may also be particularly beneficial in cancer outcomes. Studies have confirmed that marital status influences survival in a range of indications including nasopharyngeal carcinoma, osteosarcoma, glioma and rectal cancer^6–9^. However, few studies have investigated the correlation between marital status and survival in patients with mPDAC.

Nomograms have are effective and reliable tools that can be used to predict patient outcomes and inform clinical decision making^10–12^. Previous research has established nomograms to predict survival in patients with PC^13^. Recent studies have also constructed prognostic nomograms to explore the influence of marital status in bladder and metastatic lung cancer^14,15^ yet the impact of marital status on the prognosis of mPDAC remains to be determined. This study aimed to investigate the impact of marital status on the survival of mPDAC patients using data from the Surveillance, Epidemiology and End Results (SEER) database.

## Materials and methods

### Data source and study design

**Figure 1:**
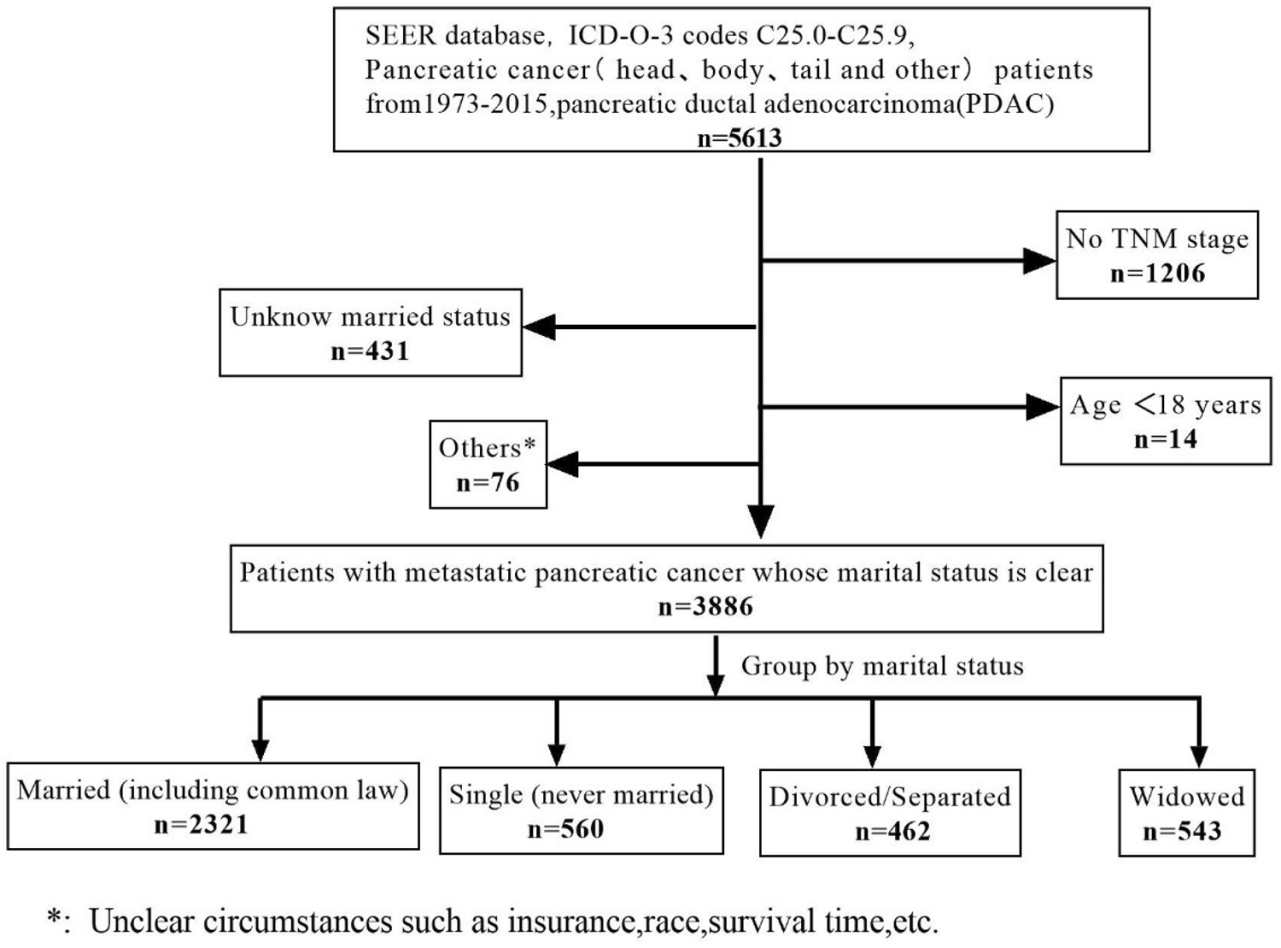
A flow diagram showing the study selection process.

The SEER database was established by the US National Cancer Institute (NCI) and stores data for population-based studies. The SEER program covers 97% of cancer types and includes data on cancer incidence, survival and mortality^16^ across several disparate geographical localities. The SEER*Stat software was used to select patients with PC from 1973 to 2015, and a total of 5613 mPDAC patients were initially identified. To screen for suitable patients, the following exclusion criteria were set: (1) age < 18 years; (2) unknown follow-up time; (3) unknown TNM information; (4) unknown tumor grade; (5) unknown marital status; (6) unknown race; (7) unknown surgery of the primary site and (8) unknown survival status. PDAC patients with distant metastasis that were classified as M1 based on AJCC 7th edition TNM system were included in the analysis. A total of 3886 eligible patients were included in the study that were randomized to primary (n = 2722) and validation cohorts (n = 1164) using the “caret” package in the R software (Fig. 1).

### Study variables

Patient variables were extracted from the cohorts including demographics (age, race and sex), marital status, year of diagnosis, geographical area, household income (country-level median household income in the past 12 months), insurance status, tumor grade, tumor size, surgery (primary site, lymph node), distant metastatic site (liver, brain, bone, lung, multiple sites and others) and survival information (time and status). The main study endpoint was overall survival (OS) that was calculated from the time of diagnosis to death due to any reason. In our study, patients were categorized as ≤ 60 and > 60 years. Patients were grouped according to marital that was defined as being married, divorced/separated, single or widowed. The patterns of metastasis were defined as liver only, brain only, bone only, lung only, multiple sites and others. Tumor sizes were defined as ≤ 30 mm, 31–49 mm and ≥ 50 mm. Insurance status was classified as any Medicaid/insured and uninsured. Surgery of the primary site was defined as complete resection, non-complete resection and no surgery.

### Statistical analyses

Categorical variables were compared using a chi-squared or Fisher’s exact test. Continuous variables were evaluated using a student’s t-test. The OS for patients with different marital status was determined by Kaplan–Meier curves and compared using a log-rank test. Univariate and multivariate cox proportional hazards regression model was used to detect the independent risk factors of OS and the parameters referred to the hazard ratio (HR) with the corresponding 95% confidence intervals (CI). The results from the Cox regression analysis were used to construct a predictive nomogram for OS at 1-, 2-, and 3 years, and the concordance index (C-index) was used to assess its performance. Larger the C-index values represent improved accuracy for prognostic prediction^17^. Concordance between the actual and predicted survival probabilities were internally and externally measured using calibration curves with 1000 bootstrap resamples. All statistical analyses were conducted using R software (version 4.0.5) or SPSS software (version 21.0), and *P*-values < 0.05 were considered statistically significant.

### Methods statement

All methods were performed in accordance with relevant guidelines and regulations. Ethical approval was also waived as the SEER database is a public data source that contains no personal identifiers.

## Results

### Patient characteristics

There were 2722 patients in the primary cohort and 1164 patients in the validation cohort. The characteristics of the patients are summarized in Table 1. Of the 2722 patients in the primary cohort, 1441 (52.9%) were male and 1281 (47.1%) female and in the validation cohort 664 (57%) patients were male and 500 (43%) patients were female. There were 1988 (73%) patients > 60 years old in the primary cohort and 852 (73.2%) patients > 60 years old in the validation cohort. The pattern of metastases for the major proportion of the patients in both cohorts was liver metastasis. Also, the majority of patients did not have surgery or lymph node dissection. In the primary cohort, 1603 (58.9%) patients were married, 398 (14.6%) patients were single, 324 (11.9%) patients were divorced or separated and 397 (14.6%) patients were widowed.

**Table 1:** Summary of the demographics and clinic-pathologic characteristics of the patients in the study.

Patient demographic or characteristic	Total n = 3886(100%)	Primary cohort n = 2722(100%)	Validation cohort n = 1164(100%)	*P*-value
**Age (years)**				0.917
≤ 60	1046 (26.9%)	734 (27.0%)	312 (26.8%)	
> 60	2840 (73.1%)	1988 (73.0%)	852 (73.2%)	
**Race**				0.527
White	3079 (79.2%)	2169 (79.7%)	910 (78.2%)	
Black	491 (12.6%)	339 (12.5%)	152 (13.1%)	
Other^a^	316 (8.0%)	214 (7.9%)	102 (8.8%)	
**Gender**				0.019
Female	2105 (54.2%)	1441 (52.9%)	664 (57.0%)	
Male	1781 (45.8%)	1281 (47.1%)	500 (43.0%)	
**Year of diagnosis**				0.013
2010–2012	1911 (49.2%)	1303 (47.9%)	608 (52.2%)	
2013–2015	1975 (50.8%)	1419 (52.1%)	556 (47.8%)	
**Grade**				0.662
Well differentiated; Grade I	306 (7.9%)	214 (7.9%)	92 (7.9%)	
Moderately differentiated; Grade II	1436 (40.0%)	1000 (36.7%)	436 (37.5%)	
Poorly differentiated; Grade III	2050 (52.8%)	1447 (53.2%)	603 (51.8%)	
Undifferentiated; Grade IV	94 (2.3%)	61 (2.2%)	33 (2.8%)	
**Tumor size (mm)**				0.600
≤ 30	939 (24.2%)	649 (23.8%)	290 (24.9%)	
31–49	1554 (40.0%)	1102 (40.5%)	452 (38.8%)	
≥ 50	1393 (35.8%)	971 (35.7%)	422 (36.3%)	
**Surgery of primary site**				0.950
No	3459 (89.0%)	2421 (88.9%)	1038 (89.2%)	
Complete resection	65 (1.7%)	45 (1.7%)	20 (1.7%)	
Non-Complete resection	362 (9.3%)	256 (9.4%)	106 (9.1%)	
**Surgery of lymph node**				0.502
No	3411 (87.8%)	2383 (87.5%)	1028 (88.3%)	
Yes	475 (12.2%)	339 (12.5%)	136 (11.7%)	
**Metastasis pattern**				0.122
Liver only	2288 (58.9%)	1589 (58.4%)	699 (60.1%)	
Brain only	4 (0.1%)	3 (0.1%)	1 (0.1%)	
Bone only	48 (1.2%)	40 (1.5%)	8 (0.7%)	
Lung only	298 (7.7%)	205 (7.5%)	93 (8%)	
Multiple sites	536 (17.8%)	365 (13.4%)	171 (14.7%)	
Other^b^	712 (14.3%)	520 (19.1%)	192 (16.5%)	
**Marital status**				0.286
Married	2321 (59.7%)	1603 (58.9%)	718 (61.7%)	
Single	560 (14.4%)	398 (14.6%)	162 (13.9%)	
Divorced/separated	462 (11.9%)	324 (11.9%)	138 (11.9%)	
Widowed	543 (14%)	397 (14.6%)	146 (12.5%)	
**Household income, $**				0.785
≤ 3000	29 (0.7%)	22 (0.8%)	7 (0.6%)	
6000–8000	3350 (86.2%)	2344 (86.1%)	1006 (86.4%)	
≥ 8000	507 (13.1%)	356 (13.1%)	151 (13%)	
**Geographical area**				0.358
East area	1337 (34.4%)	949 (34.9%)	388 (33.3%)	
Western area	2549 (65.6%)	1773 (65.1%)	776 (66.7%)	
**Insured status**				0.256
Any medicaid/insured	3777 (97.2%)	2651 (97.4%)	1126 (96.7%)	
Uninsured	109 (2.8%)	71 (2.6%)	38 (3.3%)	

^a^American Indian/AK Native, Asian/Pacific Islander; ^b^Para-aortic lymph nodes, duodenum lymph nodes, peripancreatic lymph nodes, etc.

### The effect of marital status on overall survival

**Figure 2:**
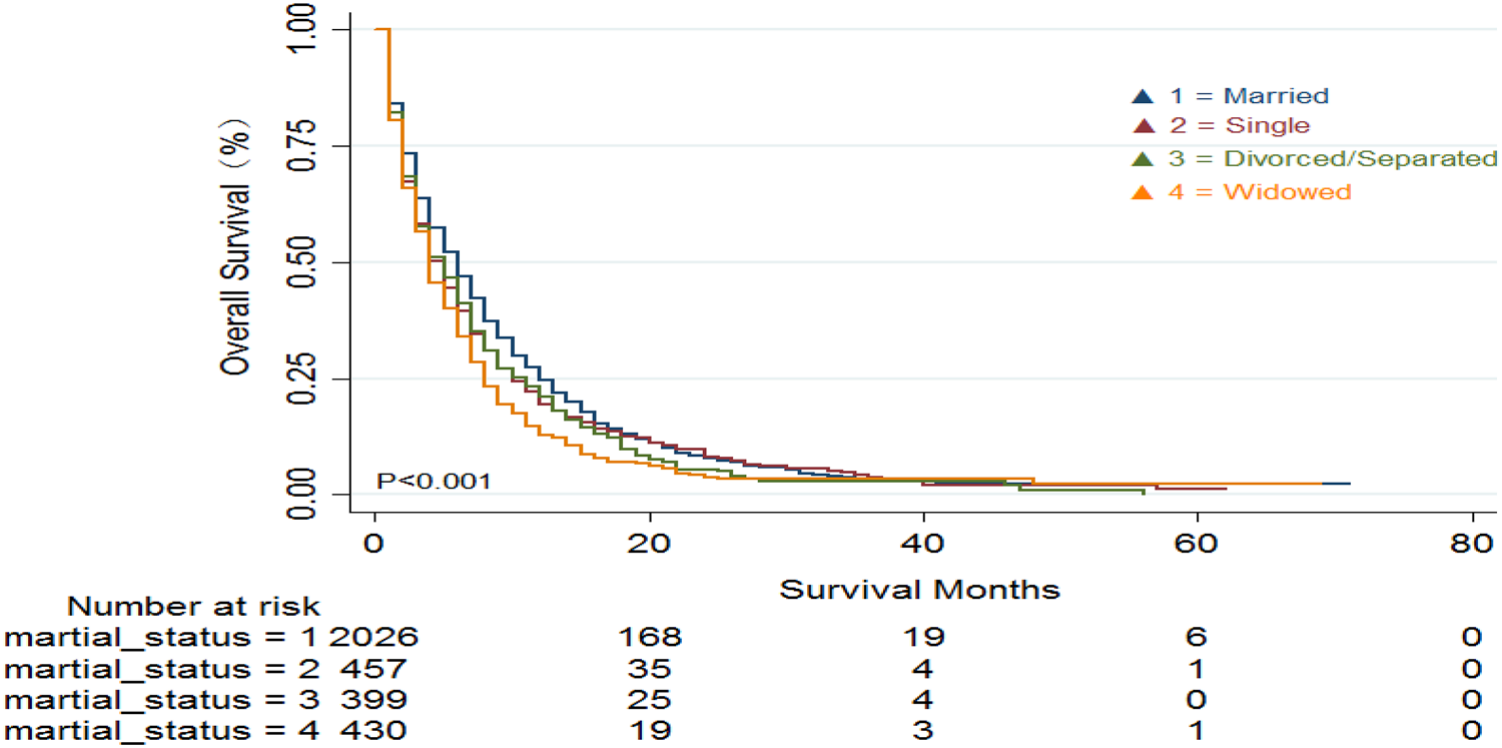
Kaplan–Meier survival analysis of overall survival (OS) in mPDAC patients of different marital status.

**Figure 3:**
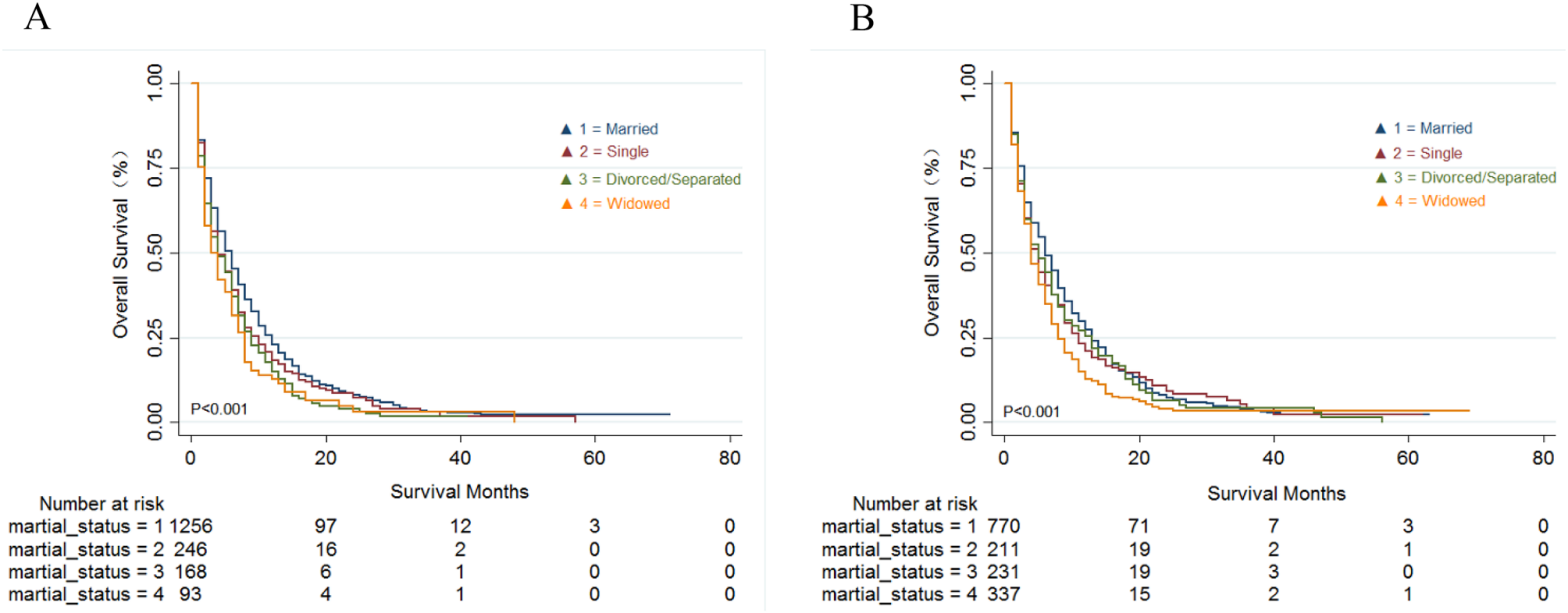
Kaplan–Meier survival analysis of overall survival (OS) in male (**A**) and female (**B**) mPDAC patients with different marital status.

As presented in Fig. 2, the Kaplan–Meier analysis was performed to compare OS in the married group of patients with the other groups. All of the mPDAC patients showed differences in OS for patients with different marital status (*P* < 0.001). The best prognosis was observed in the married group (median OS: 5 months, 95%CI: 4.589–5.411), followed by the divorced/separated group (median OS = 4 months, 95%CI = 3.396–4.604). The poorest survival was observed in the single patients (median OS = 3 months, 95%CI = 2.407–3.593) and widowed group (median OS = 3 months, 95%CI = 2.545–3.455). Kaplan–Meier survival curves were used to analyze the marital status between different genders in the mPDAC patient cohort. As shown in Fig. 3, there were also notable differences in OS for patients with different marital status irrespectively of gender (*P* < 0.001) with the highest survival in married patients and the worst survival in widowed patients.

**Figure 4:**
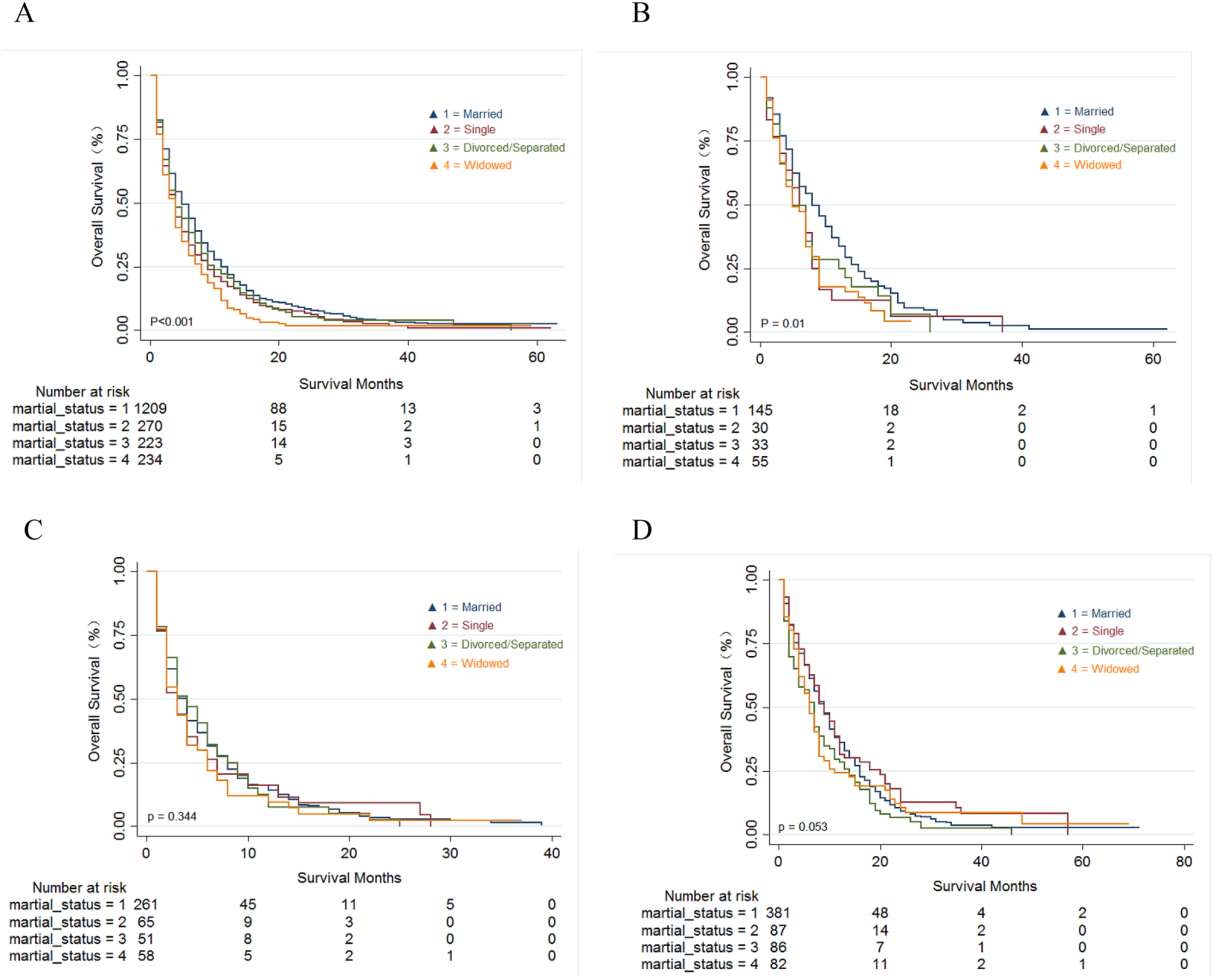
Kaplan–Meier survival analysis of overall survival (OS) in PDAC patients with different marital status with only liver metastasis (**A**), only lung metastasis (**B**), metastases at multiple sites (**C**) and other lymph node metastases (**D**).

To evaluate the effect of marital status on OS and metastasis patterns, we further conducted Kaplan–Meier survival curves in the PDAC patient cohort with different metastasis sites. As shown in Fig. 4, differences in OS were observed amongst patients of different marital status who had only liver (*P* < 0.001) and lung metastasis (*P* = 0.01). Also, married patients had the highest survival. However, no significant differences in OS were observed for different marital status in PDAC patients with metastases at multiple sites (*P* = 0.344) and those with other metastases (*P* = 0.053).

### Independent prognostic factors in mPDAC

In the primary cohort, univariate analysis of OS showed that age, grade, tumor size, surgery of the primary site, surgery of the lymph node, the pattern of metastasis and marital status were significant prognostic factors in mPDAC patients. Furthermore, factors with *P* < 0.05 from univariate analysis were incorporated into multivariate Cox analysis. Our results showed that age, grade, tumor size, surgery of the primary site, surgery of the lymph node, the pattern of metastasis and marital status were independent predictive factors for OS in mPDAC patients (Table 2).

**Table 2:** The Cox proportional hazards regression model analysis of overall survival (OS) in the primary cohort.

Variables	Univariate analysis			Multivariate analysis		
	HR	95%CI	*P*	HR	95%CI	*P*
**Age**						
≤ 60	1			1		
> 60	1.333	1.216–1.461	< 0.001	1.327	1.206–1.460	< 0.001
**Race**						
White	1			–		
Black	1.101	0.973–1.245	0.126			
Others^a^	0.988	0.852–1.145	0.871			
**Gender**						
Male	1			–		
Female	0.967	0.892–1.048	0.411			
**Year of diagnosis**						
2010–2012	1			–		
2013–2015	1.004	0.925–1.089	0.931			
**Grade**						
Grade I	1			1		
Grade II	1.065	0.906–1.252	0.445	1.076	0.915–1.266	0.375
Grade III	1.508	1.289–1.764	< 0.001	1.448	1.235–1.697	< 0.001
Grade IV	1.259	0.927–1.711	0.141	1.128	0.829–1.536	0.442
**Tumor size**						
≤ 30	1			1		
31–49	1.077	0.971–1.195	0.162	1.022	0.921–1.135	0.680
≥ 50	1.221	1.098–1.358	< 0.001	1.232	1.106–1.372	< 0.001
**Surgery of primary site**						
No	1			1		
Complete resection	0.392	0.275–0.557	< 0.001	0.566	0.375–0.852	0.006
Non-complete resection	0.489	0.423–0.566	< 0.001	0.751	0.586–0.962	0.023
**Surgery of lymph node**						
No	1			1		
Yes	0.484	0.424–0.551	< 0.001	0.710	0.563–0.894	0.004
**Metastasis pattern**						
Liver only	1			1		
Brain only	1.678	0.540–5.209	0.371	1.584	0.508–4.938	0.427
Bone only	0.749	0.533–1.053	0.096	0.872	0.619–1.227	0.430
Lung only	0.840	0.718–0.982	0.028	0.825	0.704–0.967	0.017
Multiple sites	1.242	1.102–1.398	< 0.001	1.129	1.002–1.273	0.047
Others^b^	0.694	0.623–0.774	< 0.001	0.774	0.692–0.864	< 0.001
**Marital status**						
Married	1			1		
Single	1.153	1.023–1.299	0.019	1.183	1.048–1.336	0.007
Divorced/separated	1.152	1.015–1.308	0.029	1.171	1.031–1.330	0.015
Widowed	1.485	1.323–1.668	< 0.001	1.376	1.222–1.549	< 0.001
**Household income**						
≤ 3000	1			–		
6000–8000	0.767	0.499–1.179	0.227			
≥ 8000	0.742	0.477–1.154	0.185			
**Geographical area**						
East area	1			–		
Western area	0.928	0.853–1.01	0.083			
**Insured status**						
Any medicaid/insured	1			–		
Uninsured	1.130	0.883–1.446	0.331			

^a^American Indian/AK Native, Asian/Pacific Islander; ^b^Para-aortic lymph nodes, duodenum lymph nodes, peripancreatic lymph nodes, etc.

### Prognostic nomogram for OS

**Figure 5:**
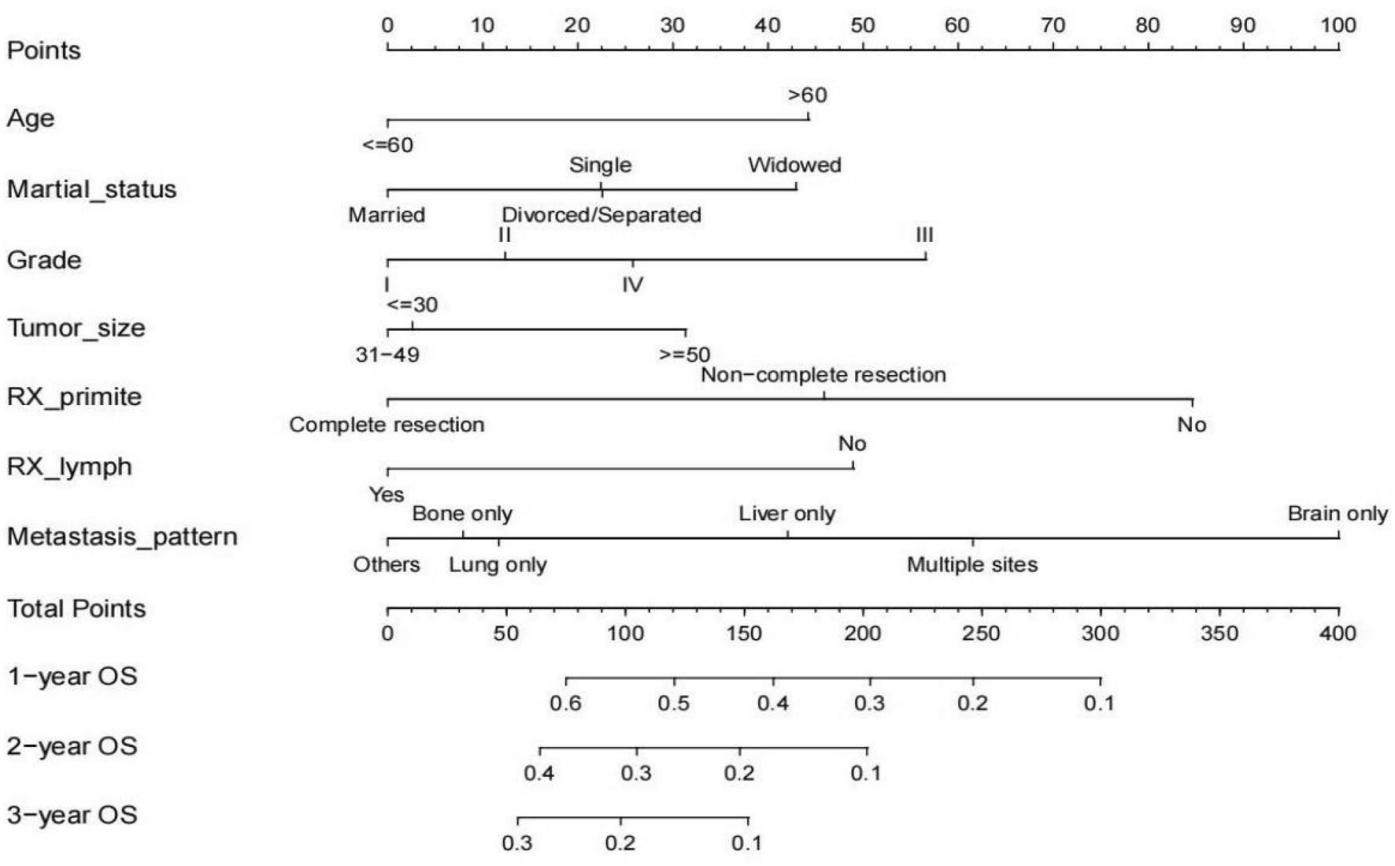
The prognostic nomograms for the OS of mPDAC patients in the primary cohort.

Based on the results of Cox multivariate regression analyses in the primary cohort, a nomogram integrating all of the vital independent factors was built to predict OS at 1, 2, and 3 years (Fig. 5). This model indicated that the metastasis pattern had the largest impact on prognosis followed by surgery of the primary site. Other factors including age, marital status, grade, tumor size and surgery of the lymph node had a moderate influence on OS. The specific scoring system of the nomogram is shown in Fig. 5.

### Nomogram validation

**Figure 6:**
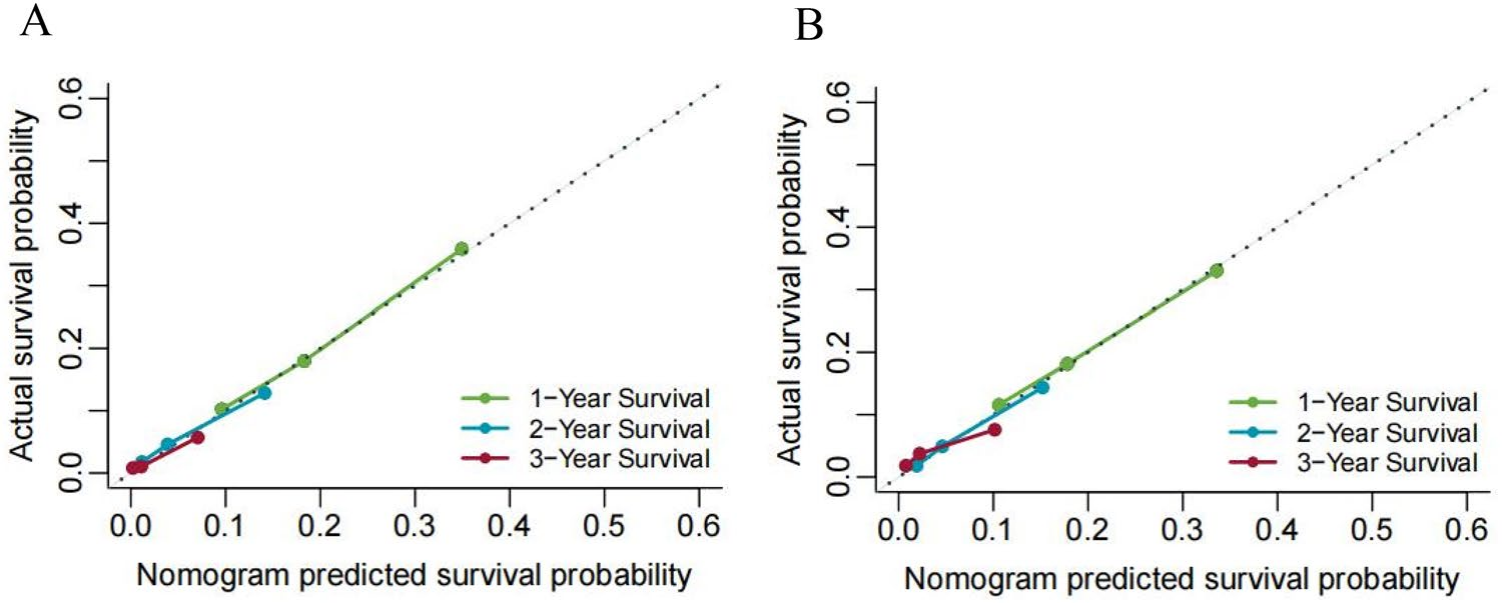
The calibration curves of the nomograms for overall survival (OS) predictions at 1 , 2 and 3 years in the primary (**A**) and validation cohorts (**B**).

The internal validation illustrated that the nomogram could accurately predict OS with a C-index of 0.633 (95%CI = 0.625–0.640). Similarly, the C-index was 0.619 (95%CI = 0.608–0.630) in the external validation. As indicated in the calibration plots, there was optimal consistency between the nomogram-predicted and the actual survival at 1, 2, and 3 years in both cohorts (Fig. 6).

## Discussion

PC is a highly lethal disease that has a 5-years survival rate of 5%^3^. However, mPDAC has an even worse prognosis. A recent study found that nearly half of the patients with mPDAC survived for less than 2 months^18^. For most patients with mPDAC, current treatments do not have favorable therapeutic efficacy and fail to prolong survival by more than a few months. Whilst conventional clinical factors such as age, tumor size, tumor staging, surgical resection and tumor radiotherapy are known to impact patient survival, we observed that marital status is also an important prognostic factor that is closely related to short and long-term survival outcomes. Similarly, Baine et al.^19^ confirmed that marital status is an independent prognostic factor that affects perioperative and long-term survival in PC patients. However, the study considered unmarried patients as the single group and did not differentiate between groups of other marital status. Our study for the first time links various marital status with the survival rate of mPDAC to explore their internal relationships.

In this study, we used the SEER database to identify differences in OS in patients with different marital status (*P* < 0.001) and found the highest level of survival in the married group. Previous hypotheses have suggested that delays in diagnosis with advanced tumors can result in poor prognosis in unmarried patients. However, a recent study reported that psychosocial factors also play a vital role in survival related to marital status that is independent of tumor heterogeneity and treatment characteristics^20^. It is known that marital status can impact psychological conditions and the general health of individuals. Married patients have fewer emotional consequences such as distress and depression compared to other patients when they are diagnosed with cancer as their partners may share part of the emotional burden^21,22^. Compared to unmarried patients, the beneficial effects of marriage on health are associated with a higher quality of life, greater financial resources and wider social support^23^. Also, married patients may prefer to receive better treatment due to decreased psychological burden and increased emotional support from their spouse that can help to maintain a lifestyle.

In this study, we also explored the influence of gender on survival rates in different marital states. Our data showed notable differences in OS for patients with different marital status regardless of gender (*P* < 0.001). The highest survival was observed in the married population, whilst the worst survival was in widowed patients. These data may be due to widowed patients having a lack or absence of emotional and financial support and reduced medical compliance. Concerning the patterns of metastasis, significant differences in OS were observed in PDAC patients with liver and lung metastasis but not in patients with metastases in multiple sites. These data may be due to the liver, lung and peritoneum being the most common metastatic sites for advanced PDAC^24^. However, PDAC patients with multiple metastases at the end-stage of disease are prone to death and are not be easily affected by other factors.

Multivariate analysis showed that marital status was an independent prognostic factor for OS in patients with mPDAC. Also, from the univariate analysis using marriage as the reference, the HRs of single, divorced/separated and widowed patients were 1.153 (1.023–1.299, *P* = 0.019), 1.152 (1.015–1.308, *P* = 0.029), 1.485 (1.323–1.668, *P* < 0.001), respectively. In multivariate analysis using marriage as the reference, the HRs of single, divorced/separated and widowed patients were 1.183 (1.048–1.336, *P* = 0.007), 1.171 (1.031–1.330, *P* = 0.015), 1.376 (1.222–1.549, *P* < 0.001), respectively. Our data confirmed that marriage is associated with a better prognosis in mPDAC patients and those patients who are divorced/separated and widowed may be at an increased risk of death.

Li et al.^25^, reported that loss of social support and the failure of response to stress in widowed patients may be a cause of higher mortality and so supportive interventions targeting unmarried people are likely to prolong survival rates. Specifically, physicians should adequately evaluate the psychological status of unmarried patients and when abnormal psychology is found, the patient should be referred to mental health experts and be given emotional care. These measures may improve clinical outcomes for unmarried cancer patients. Psychological interventions and social support should be given as part of systematic treatment to reduce the significant survival differences associated with marital status.

Nomograms are useful tools access to predict prognosis^26,27^. Recently, several nomograms have been established for the prognosis prediction of PC patients^28,29^. However, few studies have developed a prognostic nomogram for mPDAC. Based on the SEER database, we constructed a prognostic nomogram to explore the probability of 1, 2, and 3 years OS in mPDAC patients. Moreover, a C-index and calibration curves were also generated to test the predictive accuracy of this model. The C-index of the primary and validation cohorts were 0.633 and 0.619, respectively, demonstrating the optimal consistency between the predicted and actual outcomes. This nomogram identified that the metastasis pattern had the largest impact on prognosis and that marital status had a moderate influence on OS. Our model was based on data from a large number of patients that improved the accuracy of the nomogram. Also, the nomogram indicated that seven variables reflected the general status of patients and disease characteristics which is beneficial to provide doctors with valuable information for mPDAC in selecting optimum treatments.

Whilst this study provided an in-depth analysis of the relationship between marital status and prognosis in patients with mPDAC, several potential limitations still exist. Firstly, the marital status of some patients may change after registration and may contribute to different results. Secondly, the quality of the marriage may also influence the survival of mPDAC patients and psychological scores underlying the link between marriage and cancer outcomes yet this information is not available in the SEER database. We strongly suggested that future studies should include or establish a relevant scoring system, such as a psychological score, to quantify the impact of marriage on cancer and better determine the relationship between marriage and cancer prognosis. Finally, some selection biases may exist as this was a retrospective study based on the SEER database. Also, no data on therapies such as radiotherapy or molecular targeted therapy were supplied by the SEER database.

In summary, these data indicate significant differences in the OS of mPDAC patients of different marital status. Married patients had better survival compared to unmarried patients. Marital status was identified as an independent prognostic factor for OS and had a moderate influence on predicting OS. A prognostic nomogram based on multivariate analyses to predict survival for mPDAC patients was constructed and validated. This approach can be used to provide individualized survival predictions for mPDAC patients.

## Data Availability

The datasets used during the current study are available from the corresponding author on reasonable request.
